# Lessons learned from implementing a national infrastructure in Sweden for storage and analysis of next-generation sequencing data

**DOI:** 10.1186/2047-217X-2-9

**Published:** 2013-06-25

**Authors:** Samuel Lampa, Martin Dahlö, Pall I Olason, Jonas Hagberg, Ola Spjuth

**Affiliations:** 1 SNIC-UPPMAX, Uppsala University, PO Box 337, SE-751 05, Uppsala, Sweden; 2 Science for Life Laboratory, Uppsala University, Husargatan 3, SE-751 23, Uppsala, Sweden; 3 Evolutionary Biology Centre, Uppsala University, Norbyvägen 18D, SE-752 36, Uppsala, Sweden; 4 Department of Pharmaceutical Biosciences, Uppsala University, SE-751 24, Uppsala, Sweden

**Keywords:** Next-generation sequencing, Infrastructure, High-performance computing, Bioinformatics, Genomics, Data analysis

## Abstract

Analyzing and storing data and results from next-generation sequencing (NGS) experiments is a challenging task, hampered by ever-increasing data volumes and frequent updates of analysis methods and tools. Storage and computation have grown beyond the capacity of personal computers and there is a need for suitable e-infrastructures for processing. Here we describe UPPNEX, an implementation of such an infrastructure, tailored to the needs of data storage and analysis of NGS data in Sweden serving various labs and multiple instruments from the major sequencing technology platforms. UPPNEX comprises resources for high-performance computing, large-scale and high-availability storage, an extensive bioinformatics software suite, up-to-date reference genomes and annotations, a support function with system and application experts as well as a web portal and support ticket system. UPPNEX applications are numerous and diverse, and include whole genome-, *de novo*- and exome sequencing, targeted resequencing, SNP discovery, RNASeq, and methylation analysis. There are over 300 projects that utilize UPPNEX and include large undertakings such as the sequencing of the flycatcher and Norwegian spruce. We describe the strategic decisions made when investing in hardware, setting up maintenance and support, allocating resources, and illustrate major challenges such as managing data growth. We conclude with summarizing our experiences and observations with UPPNEX to date, providing insights into the successful and less successful decisions made.

## Review

Molecular biology has in the last couple of years seen an immense growth in experimental data, with perhaps the largest contributor being next-generation sequencing (NGS). With constantly increasing throughput, these technologies have transformed molecular biology into a data-intensive field that presents new challenges in storing and analyzing the huge volumes of data generated [1,2]. As biological sequencing continues to grow exponentially, bioinformatics has emerged as a key discipline to manage and analyze this data [3].

The computational power of desktop computers is insufficient for the analysis of today’s biological data sets and scientists are dependent on high-performance computing (HPC) and large-scale storage infrastructures [4,5]. As the price per sequenced base is decreasing faster than computers are increasing in computational power [6], it is not possible to simply wait for faster computers to resolve the situation. Bioinformatics tools for processing and analyzing data from NGS are relatively new, and in many cases not well adapted for HPC. There are many specialized tools for different tasks, creating the need for frameworks that integrate such tools into easy to use pipelines [7-12].

Apart from computing power and software tools, a big challenge in molecular biology is how to store the generated data. Scientists are reluctant to discard raw data since improved algorithms may help extract further information from them in the near future. The steps of NGS analysis also generate large temporary files, and it is not uncommon for projects to require 5-10 times as much storage during the analysis phase as required by the initial raw data itself. With multiple compute nodes, as is common in HPC, comes the need to share data between the nodes, which also adds to the complexity [13]. Further, many journals require the final datasets to be made publicly available in order for manuscripts to be published [14,15]. Long term archiving of large amounts of data is not a trivial task, and it is evident that the NGS community is facing a storage problem [16].

A researcher who wants to use NGS technologies needs extensive IT and bioinformatics expertise or access to specialists with these skills as well as access to a high-performance infrastructure for analyzing and storing the generated and analyzed data. However, the IT expertise to provide these solutions is not usually available to the average biology research group, which requires the group to either bring an expert into the group or outsource.

In this paper we present a Swedish infrastructure, UPPNEX, aimed at meeting these challenges by providing a high-performance cluster and storage system equipped with an actively maintained bioinformatics software suite, as well as application experts to assist with bioinformatics analysis.

### Next-generation sequencing in Sweden

Sweden has a long tradition in biological sciences, such as gene sequencing and methods development, and in recent years, an active NGS community has emerged. Initially, several small *sequencing platforms* were formed around the larger universities of Sweden to serve nearby researchers. In 2010, Science for Life Laboratory (SciLifeLab) was founded as a cooperation between four universities in the Stockholm-Uppsala region of Sweden: Karolinska Institutet, Royal Institute of Technology, Stockholm University and Uppsala University. This initiative included large investments in NGS technologies and the national sequencing platforms within SciLifeLab, which today consists of eight Illumina machines [17] (HiSeq2000, HiSeq2500, MiSeq), ten from Applied Biosystems [18] (Solid 5500xl, Solid 5500xl Wildfire, Ion Torrent, Ion Proton) and three 454 Life Sciences (Roche) [19] (GS-FLX). Apart from the 21 instruments owned by SciLifeLab, there are at least five other instruments available at the larger universities of Sweden. In addition, apart from performing the actual sequencing, SciLifeLab also assists with the data analysis and interpretation; either as a collaborative project or as fee-for-service. SciLifeLab bioinformaticians typically take care of running the data through a standardized pipeline where the most common preparatory steps of NGS analysis are carried out, such as cleaning up the data and aligning short reads to a reference genome [20]. After this initial step, the researchers are free to continue with any custom pipelines or analyses, based on the prepared data.

In the early days of sequencing in Sweden, data and results were generally delivered to clients on external hard disks. This was not only cumbersome, but also impractical as projects increased both in size and numbers. There was a clear need for an infrastructure that could deal with large quantities of data and provide HPC resources for analysis, tightly coupled with the data storage.

In order to tackle these growing challenges, a national resource for NGS analysis *“UPPMAX cluster and storage for next-generation sequencing”* (UPPNEX), was established, and enabled by a strategic grant in 2008 by the Knut and Alice Wallenberg foundation (KAW) [21] together with the Swedish National Infrastructure for Computing (SNIC) [22]. Formally, UPPNEX is a project at Uppsala Multidisciplinary Center for Advanced Computational Science (SNIC-UPPMAX), which is one of six SNIC centers in Sweden and Uppsala University’s resource for HPC, large-scale storage and related know-how. The objective of UPPNEX is to provide computing and storage resources for the NGS community of Sweden, together with an infrastructure of software, tools and user support. The services are provided free of charge for Swedish academia and resources are allocated to projects on the basis of estimated requirements, with sequencing platforms having a higher priority. The sequencing platforms within SciLifeLab deliver data to UPPNEX projects and over the last few years many prominent research projects involving NGS have been performed with UPPNEX resources [23-30]. Below, we describe the implementation of the infrastructure, outline architectural choices and strategic decisions made when implementing UPPNEX and follow up with current activities and lessons learned.

## UPPNEX infrastructure

### Data flow

**Figure 1:**
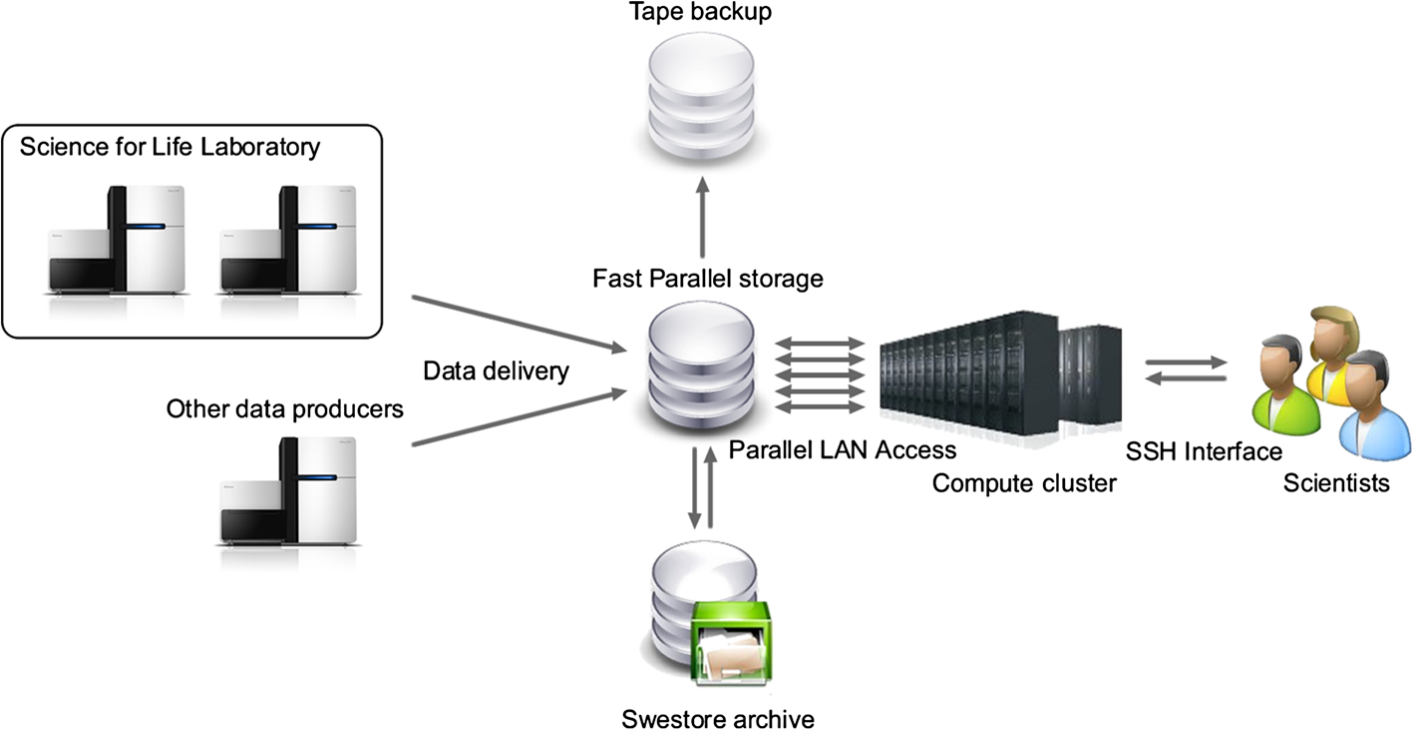
**Overview of the architecture of the UPPNEX infrastructure.** Data is produced by sequencing platforms or individual research groups, and transferred to UPPNEX. Research groups then log in to the UPPNEX system and analyze their data using the installed software. For long-term storage, there is a possibility to move data from UPPNEX to the Swedish national storage initiative SweStore.

The UPPNEX data flow is described in Figure 1. After sequencing, the platforms transfer data to UPPNEX storage via a dedicated server using Rsync [31]. Sufficient bandwidth for transferring the data from the sequencing machines to UPPNEX storage is provided by a 10Gbit ethernet connection to the Uppsala University backbone further connected to the fast Swedish national university backbone SUNET [32,33]. The platform staff then runs initial analyses and preparatory processing of the data, whereafter the final sequence data are delivered to the respective UPPNEX projects into a dedicated “inbox”.

A national storage initiative, SweStore [34], provides long term storage, mirrored on at least two different SNIC centers. After an analysis has completed, the data and results can be moved to SweStore where it can be stored temporarily or archived for longer periods. UPPNEX uses iRODS [35] to facilitate moving data between different types of storage resources.

### Hardware

When UPPNEX was to invest in resources for computing and storage, two strategic decisions were made: *i*) UPPNEX computational resources would form part of a larger investment, resulting in a cluster shared with other scientific domains; and *ii*) UPPMAX would provide one large file system for all resources. The decision to be part of a larger investment was motivated by the fact that it would result in a better price and because UPPNEX usage was estimated to be low at the beginning and increase over time, so that idle UPPNEX resources could then be utilized by other users. A single file system provides a simplified user experience with a unified view of the file system regardless of what cluster the user connects to. The only significant drawback is that if the file system fails, it would render all computational resources unavailable.

UPPNEX took part in the procurement of a general-purpose computational cluster for UPPMAX: an HP cluster consisting of 348 nodes, where each node was equipped with 8 cores for 2,784 cores in total. The majority of the nodes were equipped with 24 GiB of memory but a few *fat nodes* had more RAM: 16 with 48 GiB and 16 with 72 GiB. For storage, a parallel file system of 462 TiB usable space, was purchased from Panasas. UPPNEX’s part of the purchase originally amounted to 900,000 CPU core hours per month and 339 TiB usable space of the parallel file system directly connected to the cluster. After two years (in 2011) the parallel storage was expanded with another ca 416 TiB usable space, fully dedicated to UPPNEX, making UPPNEX storage reach approximately 755 TiB. This does not include users’ personal global scratch folders, which have been heavily used, with many UPPNEX users having global scratch quotas of a few terabytes. Thus the true amount of NGS data on parallel storage lies between the 755 TiB dedicated to UPPNEX, and the total 878 TiB. Around the same time, UPPNEX’s share of the cluster was increased to 1,184,000 CPU core-hours per month. Storage bandwidth is mainly limited by the network interfaces, which is 1 gigabit per second per compute node and 10 gigabit per second per rack (containing 44 compute nodes). Bandwidth has not been a problem for most users, but metadata performance has been much more problematic, at least with the first generation of storage (constituting the first 462 TiB). The additional 416 TiB were of a newer generation, with more RAM dedicated to metadata operations, which has resolved the problem. The system is complemented with a tape storage system to back up all home folders and non-temporary project data.

In 2011, the resources at UPPMAX were complemented with a Symmetric Multi-Processor machine (SMP), with 64 cores and 512 GiB of memory, which was soon upgraded to 2 TiB. This resource is used primarily for *de-novo* assembly computations, which typically require large amounts of RAM for keeping data structures, for example, De Bruijn graphs [36], in memory.

A single job queue was implemented in the Simple Linux Utility for Resource Management (SLURM) [37] resource management system, where projects get a limited number of priority-computing hours per month. After these priority-hours are spent, project members can still submit jobs, but with a lower priority.

### Software

A big undertaking for an NGS e-infrastructure is the installation and maintenance of the wide and rapidly evolving ecosystem of software required for analysis. UPPMAX system experts (system administrators) and application experts assist users with installing required software. In many cases this has demanded substantial effort since many NGS software applications are not prepared to run on multi-user, multi-project HPC systems. UPPMAX organizes software using a module system [38] to set up specific environments for tools. Installed bioinformatics software include alignment programs (e.g., BWA [20], Mosaik [39], Bowtie [40], Tophat [41], MAQ [42], BioScope and LifeScope [43]), *de novo* assembly software (e.g., Abyss [44], Velvet [45], Mira [46]), various downstream analysis programs (e.g., Cufflinks [47], MrBayes [48], SAMtools [49], Annovar [50]) and general tools (e.g., BioPerl [51], Picard [52], GATK [53]). Reference genomes are also available locally at UPPNEX and these are continuously updated by the application experts.

The installation and maintenance of software is done in the following way: Initially, users are encouraged to compile and install software themselves in their home folders. Users who find this too difficult can ask for the software to be installed by UPPNEX staff and if the software is deemed interesting for several users, the request will be taken care of by a system expert and/or application expert. Typically the person who installed the software will take care of upgrades, when an upgrade is requested in the support ticket system [54].

Most of the installed software at UPPNEX is only available via command line interface. This is not ideal for all users, and UPPNEX has an ongoing project to implement graphical user interfaces for these tools as an alternative. UPPNEX security policy does not permit public web servers to be connected to the file system, which limits the usefulness of web-based GUI tools for UPPNEX. The workflow tool Galaxy [7-9] has been installed, but users are currently limited to running their own instances over an SSH-tunnel. A single, central instance would be desirable from the users point of view as it facilitates the sharing of data and workflows but this would require dedicating a part of the storage system to Galaxy, which has not been feasible with the steadily increasing data volumes at UPPNEX.

### User support

Almost from the beginning, UPPMAX focused on the use of *application experts* to establish a vital link between users and the HPC systems. New users often lack experience with the operating systems and tools used in HPC. This applies especially to biology and NGS, where the exponentially diminishing cost of sequencing allows virtually every researcher to create huge amounts of data requiring HPC for analysis [55]. At UPPMAX, around ten researchers are employed part-time as application experts in various fields, helping users with tasks both common and novel. The support may be administered officially through the support ticket system or unofficially through personal communication with users, UPPMAX introduction days and courses. There are several similar support models, where application experts who are not employed directly by the research groups performing the analysis, have been established in Sweden and they differ mostly in the duration and depth of support they offer, listed here shortest to longest: 

**Biosupport.se **[56]**:** an online support forum moderated by a staff of bioinformaticians.

**BILS **[57]**:** An organization giving bioinformatics support for free for up to two weeks, established to address the need for bioinformatics analysis in Swedish academia.

**WABI **[58]**:** A new organization that will work as bioinformatics consultants in research groups for longer periods of time.

Contact between the UPPMAX application experts and system administrators is facilitated by weekly meetings, as well as via email, phone and common access to the support ticket system.

UPPNEX is also involved in organizing courses for biologists in basic Linux usage, how to run the most widely used analysis tools and how to manage data on a compute cluster. These courses are given a couple of times per year and have so far been very popular.

### Pipelines

UPPNEX is being used by the three sequencing platforms, each with their own data delivery workflow. One platform generally runs a comprehensive pipeline consisting of quality control, alignment, SNP calling and SNP effect prediction [59] on most of the samples sequenced and finally presents the results in a Galaxy instance for the customer to view. Another platform mostly uses Applied Bioscience machines and performs similar analyses using LifeScope [43]. The third platform uses a Perl-based pipeline developed in-house.

## Results and discussion

### Storage

**Figure 2:**
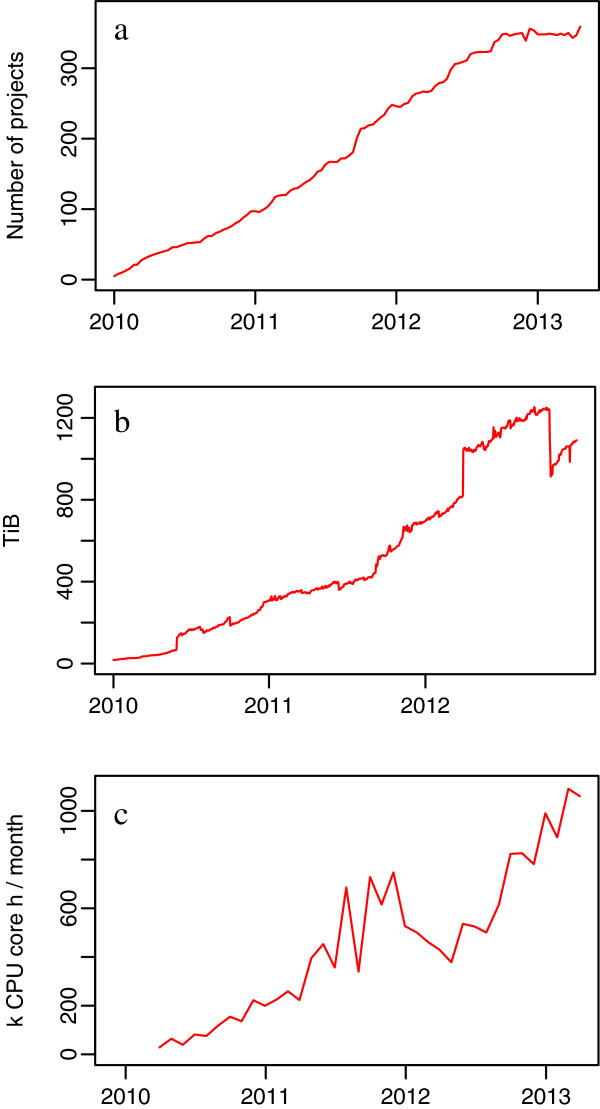
**Overview of the resource utilization at UPPNEX.a** Number of active projects. The number of active projects has steadily increased, although a plateau seems to have been reached. **b** Total amount of NGS storage consisting of fast parallel storage, global scratch folders, and NGS storage on SweStore. Storage has generally increased with some small dips associated with clean-up campaigns. The larger deviation in 2012 is believed to be due to data duplication during migration to a new storage system. **c** CPU usage trend. Usage has increased but with significant variations. The dip in late 2011 was due to a longer downtime at UPPMAX for move to a new computer room. The explanation for the long drop early in 2012 is unknown.

Since its inauguration in 2009, UPPNEX has displayed a roughly linear increase in the number of projects, which amounted to 357 active projects in April 2013 (see Figure 2a). Recently, a plateau seems to have been reached, where the rate of project expiration is on par with the rate of project creation. The total amount of stored NGS data has also increased steadily (see Figure 2b), but changes in the types of data stored by sequencing platforms, as well as user education on how to use scratch storage for temporary results and better use of compression of files, have greatly affected the data size on disk over time. The small dips in total NGS storage in Figure 2b are due to time points where major users and platforms were asked to clean up project folders, a process which over time has resulted in SOPs for data management. The sudden increase followed by the equally sudden decrease in 2012 is believed to be due to data duplication during migration to a new storage system. Other actions that have helped keep the amount of data low on the fast, but expensive parallel storage system at UPPMAX have been the implementation of more strict policies for allowances, cleaning up of temporary data, compressing files in inefficient file formats like raw text, and an increased use of the SweStore national storage.

### Computational resources

The usage of UPPNEX computational resources has also increased over the years, as shown in Figure 2c, and today UPPNEX is very close to reaching it’s maximum allocation of CPU core-hours per month. The low usage of HPC resources in the first year can be explained by start-up time for sequencing platforms as well as a delay when recruiting bioinformaticians and training them in HPC. The drop in late 2011 was due to a longer downtime at UPPMAX for the move to a new computer facility. The reason for the long drop early in 2012 is unknown. Sharing a cluster with other scientific domains has had advantages and disadvantages. One of the advantages has been in resource utilization — when UPPNEX did not use all available resources, researchers from other disciplines were able to use the remainder with a lower priority. However, due to the difference in job length and width (in terms of cores used), there have been cases where biologists (who typically run many narrow jobs) have complained about the wide and long jobs blocking their much more interactive use of the system. Perhaps the biggest advantage of establishing a shared cluster has been the buy-in of system administrators and HPC experts from other disciplines, which has paved the way for a well-functioning resource for bioinformatics.

Early in the NGS era, analysis software only used a single core and no parallel programming techniques, such as threading or Message Passing Interface (MPI). This has changed over the years and today many software applications use threading to speed up calculations. Eight cores per node have so far been sufficient at UPPNEX, but future resources should probably have an increased number of cores to benefit threaded programs. To date, NGS-applications using MPI to spread the load over many compute nodes rather than just on many threads, have been scarce. A problem at UPPNEX has been that users could only reserve either a core or a node, consisting of 8 cores. Since each core has only 3 GiB of accompanying RAM, users were forced to reserve a full node (24 GiB) when more than 3 GiB was needed. According to many users, this has often been the case [personal communication]. This problem has been eliminated in a recent version of the resource management system with the possibility to bind jobs to any number of CPUs on a node.

Computational tasks in life sciences often rely on huge data structures, for example, keeping entire genomes in memory and making computers with large memory capacity important. UPPNEX’s fat nodes have been extensively used and have in many cases had the longest queues. Projects that require more RAM than the fat nodes (72 GiB), such as *de novo* sequencing projects of large genomes and transcriptome assembly projects, benefit from the SMP. This SMP has enabled several large studies in Sweden, such as the genome and transcriptome assembly of the Collared Flycatcher (Ellegren *et al.*[60]), Norwegian Spruce [61], and Herring [personal communication].

In summary, having a good mix of general purpose computational power, the possibility to book an arbitrary number of cores (not just whole nodes) for jobs requiring more CPUs or memory, together with fat nodes and an SMP for very large memory requirements has proven to be a very useful and cost-effective infrastructure for NGS analysis.

### Backup

Providing backup for the large-scale data from UPPNEX is a challenging task. UPPNEX uses a tape robot for backup, where bandwidth (the amount that can be stored each day) is the limiting factor (currently around 6 TiB per day). This can cause problems, for example if users rename a folder that contains terabytes of data, since this will be interpreted by the backup software as changed data, which would quickly limit the remaining backup capacity for other projects. A seemingly reasonable solution to this was worked out by limiting the amount of data that can be backed up per day, per project, and then informing users about this limit. This limit is currently set to 800 GiB per day per project. This works in such a way that if users do something that creates 1600 GiB of new or changed data, these changes will be completely backed up after two days, if no other significant changes in the data are generated. This strategy has been well received by the UPPNEX users, and provides a way to distribute the backup capacity in a reasonably fair way.

### Software and user support

The largest undertaking of UPPNEX has been maintaining the ever-expanding NGS-software ecosystem and supporting a user community that was new to batch-processing and HPC systems. In the early days of UPPNEX, very few software packages worked well in a shared computational cluster and UPPNEX staff were required to modify some software to make it run as jobs with a resource management system. Maintaining such software with many and frequent upgrades requires structured documentation, responsibility roles, and SOPs for maintenance. Graphical user interfaces have had to be de-prioritized, due to lack of resources and because available tools are often not well adapted for shared HPC systems. However, GUI tools are especially important for many users and will be a priority for UPPNEX in the coming years.

The system- and application experts have had a pivotal role in both software maintenance and user support and at times it has been challenging to recruit the right people. UPPNEX has greatly benefited by the ability to recruit people with a mixed background, between, for example, bioinformatics and information technology, such as bioinformatics engineers with an interest in computer hardware. These people have been important links between the biology and computing communities. Establishing training schools and workshops for long-term sustainability in this area has required significant efforts.

### Users

The user base of UPPNEX consists to a large extent of bioinformaticians, but also includes biologists or computational scientists who have learned basic bioinformatics. This differs from traditional HPC users who generally are much more experienced with computers. To access the UPPNEX system, users need to use a terminal, log-in via Secure Shell (SSH) and then use Linux command line tools to submit and monitor jobs — skills that are not common among biologists. This has required substantial effort from UPPNEX over the years to educate a large number of new users, many of whom had only used graphical operating systems, such as Microsoft Windows.

### Resource allocation

It is easy to allocate resources when they are plentiful, and in the beginning, UPPNEX was generous with both storage and computational resources. As resource utilization increased, it became necessary to be more restrictive and require more efforts from users. It has been difficult to get people to clean up and remove temporary data after analysis steps, but with a stricter policy, especially on storage quota, the users also have acquired new skills in big data management with compression tools and use of scratch media for analysis. However, it is still a challenge to provide resources without a visible cost to over 350 projects and we realize that we need to improve this in order to combine an efficient service with a fair allocation policy. For example, one way we are working to achieve this is by investigating ways to automatically detect unnecessary temporary files, and files stored in inefficient file formats.

## Other NGS infrastructures

Infrastructures for analysis and storage of NGS data exist on many levels of capacity, complexity and sophistication. The most basic infrastructure needed for doing general analysis on NGS data is a large multi-threaded server, or small cluster, tightly coupled with the sequencing machine [62], though many benefits can be drawn from centralizing the resources within a university or other institution [63].

UPPNEX is not alone in building on an existing HPC infrastructure and tailoring it towards analysis and storage of NGS data. Below is a brief comparison of UPPNEX with a few infrastructures that share some characteristics.

One of the largest infrastructures with similar goals is the BioWulf cluster [64] at the National Institutes for Health (MD, USA). With its impressive 12000-plus compute cores, BioWulf is clearly much larger than UPPNEX in terms of computing power. There are similar characteristics with UPPNEX, such as the use of a resource management system, a central NFS-mounted file system, a variety of node sizes (in terms of RAM size) and a large selection of pre-installed software for NGS analysis. In contrast, BioWulf does not seem to share resources with other domains and the availability of application experts is uncertain.

Another example is the BioI team at the University of Texas [65], in conjunction with the Lonestar cluster at the Texas Advanced Computing Center (TACC) [66]. The Lonestar cluster has most commonly used NGS software installed and compared with UPPNEX, the BioI team is a user-organized community, formed to help other users in the same domain [65], whereas UPPNEX has system- and application experts who are specialists in solving problems typical for NGS analysis and storage, hired as staff.

The infrastructure which may be most similar to UPPNEX is the HPC center at the University of Florida (HPCUFL) [67]. HPCUFL offers many NGS-related packages along with software from other domains. Support is also available from application experts within molecular biology and training sessions in NGS analysis. The total amount of fast storage is similar in size to UPPNEX, but they also offer long-term storage. The main difference is that while UPPNEX provides all resources for free, users must pay HPCUFL if they wish to use more than a certain amount of CPUs. In addition, project storage folders are not located on a parallel file system at HPCUFL, which might require data transfer delays between project- and scratch storage systems.

A prominent infrastructure targeted towards the analysis of NGS data that adopts quite a different approach is GenomeSpace [68]. In contrast to the other infrastructures mentioned, GenomeSpace focuses on providing an integrated graphical user interface to popular NGS tools. By integrating the Galaxy bioinformatics platform [7], even software that typically runs on the command-line could be integrated into the environment. GenomeSpace is a rather new project and it will be interesting to see how well this architecture and strategy will perform compared to the more traditional HPC-based approach taken by UPPNEX and other organizations.

## Conclusions

UPPNEX is a mature and well functioning infrastructure for NGS analysis in Sweden and has experienced a large increase in the number of projects, amount of data and computations during its first four years of operation.

Over the years there have been many decisions made regarding the architecture and implementation of the UPPNEX project, of which some have been more successful than others. Below we summarize some of our lessons learned and conclude with an outlook of how we envision the project to evolve in the future.

### Lessons learned

#### Experienced system experts greatly ease development

As a project within an existing HPC center (UPPMAX), UPPNEX has been able to take advantage of the knowledge from system experts who have been working with HPC many years prior to the establishment of UPPNEX. This has given UPPNEX a head start as many of the HPC-related problems had already been encountered by the system experts. Large parts of the infrastructure, such as server room and cooling systems, were already in place years before the cluster was procured and having the system experts dedicating part of their time on UPPNEX has also been a great way to speed up the development.

#### Application experts are vital to link users and system staff

One of the things we immediately noticed was the usefulness of application experts. Since the system experts are focused on the hardware and operating system, it is not realistic for them to stay up-to-date with the research field-specific software of all the scientific disciplines that use UPPMAX. This is where the application experts excel, because, by working part-time as researchers, they spend a lot of time using the software in practice and are keeping up-to-date with the field. They help out with deciding which software should be installed, installing and updating software, and give useful advice to novice users. This arrangement has proven successful, and a 2011 report on quality and renewal at Uppsala University stated: *“The facility is very well run... From our observations, scientists clearly appreciate the service. Their innovation of providing “application experts” is very important and at least partially responsible for their effectiveness”*. [69] This success has resulted in an increased number of application experts at UPPMAX.

#### Three GiB of RAM per core is not enough

A complaint often heard from UPPNEX’s users is that 3 GiB of RAM per CPU core is too little. This forces users to allocate more cores than they need just to have enough memory to run their analysis. With more memory per core they would not have to occupy cores they are not using, and the resource utilization on the cluster would be better.

#### MPI is not widely used in NGS analysis

Similarly sized systems were studied before the cluster was procured to give a hint about dimensioning. What the UPPNEX staff did not know at the time was that MPI was not widely used in NGS analysis, so the InfiniBand network included in the cluster has had very limited use within NGS analysis. Thus, a more conventional and cost-effective network, would probably have served UPPNEX equally well.

#### A central file system helps keep redundancy to a minimum

Strong efforts at UPPNEX are made to keep redundant data to a minimum and keeping central versions of reference genomes and other common files has been a good way to achieve that. Here, the parallel file system has been very beneficial to avoid performance problems when many users connect to the same file.

#### Different user communities can have complementary usage patterns

Another advantage of sharing the cluster with other user groups at UPPMAX is that other scientific domains often have a different user pattern than UPPNEX users. UPPNEX users work interactively with the booked nodes to a larger extent than many other user groups at UPPMAX, and submit much shorter jobs to the resource management system. This has the effect that the majority of the jobs running at UPPMAX during the days are UPPNEX jobs, while in the evenings, jobs from other user groups at UPPMAX can use most of the cluster. If the cluster had been used only by UPPNEX users, it would not be fully occupied during the nights.

#### Storage is needed for longer periods than core-hour allocations

When looking at the lifetime of an UPPNEX project, we see that storage is needed for a longer period of time than the core-hour allocation. Most projects get an initial data delivery from a sequencing platform, run analyses on the data for a couple of months, and then concentrate on understanding the results of the analysis and resulting in storage being used for a longer time than the core-hours.

#### Getting users to share scripts is difficult

It was hoped that users encountering similar problems could benefit from sharing their experiences through the scripts they used to control NGS software. Unfortunately, getting users to share scripts is difficult. Even if stating explicitly that scripts are to be provided “as is”, users are reluctant to submit scripts for public usage. Interviews have revealed that users do not have time to add proper documentation or cleanup their code or fear that they will be harassed with questions so they avoid sharing scripts.

#### Voting might not work well for software requests

The implementation of a voting system for prioritizing software to be installed was unsuccessful. With many software installation requests it was believed that such a system would aid in decision-making, but it was canceled due to lack of votes being cast. A vote could represent the curiosity of a single user or the real needs of entire lab.

#### Few analyses require more than 256 GiB of RAM

With fat nodes only having 72 GiB RAM, the SMP has been a critical component of UPPNEX. However, a retrospective analysis of the SMP usage has revealed that few analyses have required over 256 GiB of memory, and even fewer over 512 GiB.

### Outlook

Managing data growth is perhaps the biggest challenge for UPPNEX. We are currently investigating how to extend the resources in an efficient yet cost-effective way to ensure reliable data storage for both short and long term. Access to fast storage for parallel analyses is something we will continue to prioritize, while the ability to publish and archive data and results, as well as provide easy to use graphical user interfaces to the HPC system, are other important areas that we will put efforts into. We expect that the possibility to *stage* data on different storage types will be an important way to achieve these goals. Fast parallel – and thereby also expensive – storage should not be used for long term storage where fast access is not required. The ratio between the amounts of different types of storage will likely be an important factor to get right in the coming years.

One should always be careful when making predictions based on historical data and this is especially true within the rapidly evolving field of NGS. According to the trends in Figure 2 and the fact that new sequencing technologies with higher throughput will surely emerge, it appears that the NGS community will require many more computational resources in the coming years. It seems likely that Sweden, as well as other countries, will need to make significant investments for storage and HPC resources to ensure that the NGS community has the means to analyze the vast amounts of data produced.

## Abbreviations

GiB: Gibibyte; HPC: High-performance computing; iRODS: Integrated rule oriented data system; MPI: Message passing interface; NFS: Network file system; NGS: Next-generation sequencing; SLURM: Simple linux utility for resource management; SNP: Single-nucleotide polymorphism; SNIC: Swedish national infrastructure for computing; SSH: Secure shell; TiB: Tebibyte; UPPMAX: Uppsala Multidisciplinary Center for Advanced Computational Science.

## Competing interests

The authors declare that they have no competing interests.

## Authors’ contributions

OS, SL, MD, PO, and JH have all been involved in the planning and implementation of UPPNEX. All authors have read and approved the final manuscript.
